# Key steps and barriers in the journey of patients with epilepsy through the National Healthcare System in Spain: The EPIPASS qualitative study

**DOI:** 10.1002/epi4.12984

**Published:** 2024-07-04

**Authors:** Juan José Poza, Milena Gobbo, María Palanca Cámara, FEDE, Paloma Pérez‐Domper, Ángel Aledo‐Serrano, the EPIPASS Group

**Affiliations:** ^1^ Department of Neurology Donostia University Hospital Donostia, San Sebastián Spain; ^2^ PositivaMente Psychology Centre Madrid Spain; ^3^ University Hospital La Fe Valencia Spain; ^4^ FEDE, Federación Española de Epilepsia Madrid Spain; ^5^ Angelini Pharma Barcelona Spain; ^6^ Epilepsy Unit Vithas La Milagrosa University Hospital, Vithas Hospital Group Madrid Spain

**Keywords:** diagnosis, drug therapy, follow‐up, patient management, seizure, unmet needs

## Abstract

**Objective:**

Epilepsy requires continuous medical attention from multiple healthcare specialists, specialized facilities, and community‐based care. In Spain, there is no standardized approach to epilepsy care. The aim of this study was to identify the factors impacting on the delivery of high‐quality care by exploring key steps and barriers along the patient journey through the Spanish National Healthcare System (NHS).

**Methods:**

A qualitative study was conducted using opinions and experiences of neurologists, nurses, patients, and caregivers shared in discussion meetings. Using thematic content analyses, relevant aim‐focused statements were coded according to prespecified issues in a discussion map (i.e., key steps and barriers), and sub‐coded according to emerging issues. Thematic saturation and co‐occurrence of key steps/barriers were evaluated to identify the most relevant factors impacting on the delivery of high‐quality care.

**Results:**

Sixty‐five stakeholders took part in discussion meetings (36 neurologists, 10 nurses, 10 patients, and nine caregivers). Six key steps on the patient journey were identified: emergency care, diagnosis, drug therapy, follow‐up, referral, and interventional treatment. Of these, follow‐up was the most relevant step impacting on the delivery of high‐quality patient care, followed by drug therapy and diagnosis. Emergency care was considered a hot‐spot step with impact throughout the patient journey. Communication (among HCPs and between HCPs and patients) was a barrier to the delivery of high‐quality care at several stages of the patient journey, including drug therapy, follow‐up, referral, and interventional treatment. Resource availability was a barrier for diagnosis (especially for confirmation), drug therapy (drug availability), and referral (lack of professionals and specialized centers, and long waiting lists).

**Significance:**

This is the first study capturing perspectives of four key stakeholders involved in epilepsy care in Spain. We provide an overview of the patient journey through the Spanish NHS and highlight opportunities to improve the delivery of patient‐centered care with a chronicity perspective.

**Plain Language Summary:**

Patients with epilepsy may require prolonged medical care. In Spain, care is provided by a range of specialist and non‐specialist centers. In this study, a team of Spanish neurologists, nurses, patients and caregivers identified barriers that affect the delivery of high‐quality care for patients with epilepsy at each stage of their journey through the Spanish NHS. Specific epilepsy training for healthcare providers, appropriate resources for diagnosing and treating patients, and good communication between healthcare workers and patients were identified as important factors in providing high‐quality care for patients with epilepsy.

**Key points**

- We provide an overview of the epilepsy patient journey through the Spanish NHS from the perspectives of neurologists, nurses, patients and caregivers.
- The most relevant step impacting on the delivery of high‐quality patient care is follow‐up, followed by drug therapy and diagnosis.
- Emergency care is a hot spot step, having an impact throughout the patient journey.
- Several barriers to the delivery of high‐quality care were identified, including:
  ○ Lack of specific epilepsy training of HCPs.
  ○ Availability of resources for diagnosis, patient referral, drug therapy, and interventional treatment.
  ○ Lack of communication, both between HCPs and between HCPs and patients, at nearly every step of the patient journey.

## 1 INTRODUCTION

Epilepsy is one of the most common neurological diseases, affecting 4–10/1000 people worldwide^1^ and 7–8/1000 in Spain.^2^ It is associated with a significant physical, psychological, and social burden.^3^ Up to 40% of patients with epilepsy (PWE) show neurocognitive impairment^2^ and require a caregiver for daily activities, and approximately one third of patients fail to achieve seizure freedom despite having used two or more antiseizure medications (ASMs).^2^, ^4^, ^5^ Addressing unmet needs in refractory patients is a priority as they have poorer quality of life, more comorbidities, and higher odds of premature death than seizure‐free patients.^5^

As a chronic disease, epilepsy requires ongoing medical care. Traditional care systems, designed to respond to acute illnesses and injuries, need to evolve to accommodate patients with complex needs who require support from multiple healthcare specialists, specialized facilities, and community‐based care. The Chronic Care Model (CCM), developed to deliver proactive, integrated, patient‐centered health care, has become a reference for chronically ill patients.^6^, ^7^ The CCM promotes productive interactions between informed, activated patients and coordinated, prepared, proactive healthcare professionals (HCPs) to improve functional and clinical outcomes.^8^ The Expanded CCM additionally integrates prevention efforts, social determinants of health, and enhanced community participation.^8^

Implementation of the CCM approach has differed among European countries.^8^, ^9^ In Spain, a decentralized system has been in place since 2002, with autonomous communities responsible for healthcare administration and management.^10^ Here, the CCM approach has diverged between the central administration and the different health services of the autonomous communities.^9^ The Ministry of Health developed a strategy in 2012 to address chronicity in the National Healthcare System (NHS), recognizing the lack of coordination between healthcare levels, and between the social and healthcare systems.^9^ Consequently, those with chronic conditions and activity limitations experience significant problems accessing and journeying through the NHS,^9^ and those with multiple pathologies, comorbidities or complex needs are at particular risk.

In epilepsy care, autonomous communities in Spain plan and coordinate their own resources and health services,^2^ which are delivered from low (primary care or neurology services) to high (epilepsy units or surgery‐medical units) specialization levels.^2^ After experiencing a seizure, patients seek primary care, where they are assisted by primary HCPs, or go to hospital emergency services, where they are assisted by a neurologist or pediatrician. Patients are referred to the neurologist (if available) at the primary care center for initial diagnosis and treatment. After seizure control, patients are referred back to their primary HCP for follow‐up and adverse event control. Patients with severe, difficult‐to‐control epilepsy are referred to hospital neurologic or neuro‐pediatric services and epilepsy units, depending on the hospital specialization level. There, specific tests are performed for differential diagnosis, to identify the epilepsy type, and to optimize treatment. These units usually attend to patients with refractory epilepsy and offer interventional treatments, including surgery.^2^

A recent review of epilepsy management within the Spanish NHS highlighted knowledge gaps and areas for improvement.^2^ Key findings included the lack of a strategic plan for a consensual standardized approach to epilepsy management among most regional healthcare systems in Spain,^2^ and no routine use of epilepsy clinical guidelines at international and national levels.^4^, ^11^, ^12^, ^13^ The Spanish Society of Neurology (SEN) recently updated its management recommendations through an online app,^5^ and the Epilepsy Andalusian Society (SAdE) has released the most updated guideline to reach internal consensus on common clinical situations and standardized medical activity^14^; however, neither patients nor HCPs (except neurologists) participated in the development of the SAdE updated guidelines. Other guidelines and evidence‐based recommendations focus on the specifics of diagnosis and treatment of epilepsy, but lack the chronicity perspective.^4^, ^5^, ^11^, ^12^ A greater awareness of the patient journey through the Spanish NHS should facilitate the development and implementation of management guidelines, including education in self‐care, and ensure that patient preferences are taken into account.

Here, we conducted a qualitative analysis to explore the key steps in the PWE journey through the Spanish NHS and the barriers hindering delivery of high‐quality care using perspectives from physicians, nurses, patients, and caregivers.

## 2 METHODS

### 2.1 Study design

This was a qualitative, phenomenological study using a discussion group to explore the key steps and barriers to care in the PWE journey within the Spanish NHS from the perspectives and experience of people involved in the process. Opinions and experiences of key stakeholders were collected during shared discussion group meetings, which took place between April and June 2022.

### 2.2 Participant selection and inclusion/exclusion criteria

Professionals, researchers, and patient associations performed a snowball method to identify participants using purposive sampling. Participants were included if they were aged ≥18 years and fitted one of four profiles: (1) neurologists, (2) nurses working with PWE, (3) PWE, and (4) caregivers of PWE unable to speak by themselves due to cognitive impairment or an epilepsy‐related severe mental illness. To maximize the diversity of opinions, several criteria that could impact on participants' experiences were identified and researchers ensured representation of each criterion in discussion groups (Table 1). Criteria based on gender and age were included for all participants. For neurologists and nurses, additional criteria were based on epilepsy experience (years), health specialty, and workplace. For patients and caregivers, additional criteria were based on education level, disease severity, time from epilepsy onset to study enrollment, age at epilepsy onset, presence of cognitive impairment or mental illness, and type of healthcare facility where patients were receiving treatment (Table 1).

**TABLE 1:** Characteristics of the study participants and prespecified criteria.

A. Neurologists (*n* = 36)	*N*	B. Nurses (*n* = 10)	*N*
Gender		Gender	
Male	15	Male	1
Female	21	Female	9
Age (years)		Age (years)	
<45	19	<45	5
45–55	12	≥45	5
>55	5		
Time of experience in epilepsy (years)		Time of experience in epilepsy (years)	
<5	12	<5	3
>5	24	>5	7
Health specialty		Health specialty	
General neurologist	11	General nurse	6
Epileptologist (epilepsy unit)	12	Nurse specialized in epilepsy	4
Epileptologist (local referring specialist)	8		
Epileptologist (RCSU)	5		
Workplace		Workplace	
County hospital	10	County hospital	1
Regional hospital	22	Regional hospital	6
Reference Centers, Services, and Units (RCSU)	4	Reference Centers, Services, and Units (RCSU)	3

C. Patients (*n* = 10)	*N*	D. Caregivers (*n* = 9)	*N*
Gender		Gender	
Male	4	Male	3
Female	6	Female	6
Age (years)		Age (years)	
<45	4	<45	3
45–65	5	45–65	6
>65	1	>65	1
Education level		Education level	
No education or primary education	1	No education or primary education	
High‐school and/or vocational training	6	High‐school and/or vocational training	4
University or higher education	3	University or higher education	5
Time from disease onset to study enrollment (years)		Time from patient disease onset to study enrollment (years)	
≤2	0	≤2	1
3–10	4	3–10	1
>10	6	>10	6
Severity of epilepsy		Severity of epilepsy in patient	
No episodes (controlled with drugs)	2	No episodes (controlled with drugs)	1
Occasional episodes (<1 per month and >1 per year)	3	Occasional episodes (<1 per month and >1 per year)	5
Frequent episodes (>1 per month)	4	Frequent episodes (>1 per month)	3
Age at disease onset (years)		Patient's age at disease onset (years)	
0–11	2	0–11	6
12–17	5	12–17	1
>18	8	>18	2
Patient comorbidities		Patient comorbidities	
Cognitive impairment	–	Cognitive impairment	5
Mental illness	–	Mental illness	2
Territorial distribution of health centers		Territorial distribution of health centers	
County health center	5	County health center	4
Provincial capital health center	5	Provincial capital health center	5

*Note*: Study participants included neurologists (A), nurses (B), patients with epilepsy (C), and caregivers of patients with epilepsy unable to speak by themselves due to cognitive impairment or an epilepsy‐related severe mental illness (D).

Abbreviations: *N*, number of participants; RCSU, Reference Centers, Services, and Units.

All participants signed an informed consent form before participating in the study. The study was performed in accordance with the Helsinki Declaration (as revised in 2013), the ISPE guidelines for Good Pharmacoepidemiology Practices (GPP), the General Data Protection Regulation UE 2016/679, the Standards for Reporting Qualitative Research,^15^ and the Consolidated Criteria for Reporting Qualitative Research.^16^ Furthermore, the study protocol (December 2021) was approved by the Ethical Committee of the Hospital Ruber Internacional. The final report was delivered in October 2022.

### 2.3 Discussion groups

Participants from the four profiles (neurologists, nurses, patients, and caregivers) were recruited and distributed in groups from six to 10 subjects, assuring at least one representative of each prespecified profile per group to encourage productive discussion (Table 1). Participant selection was performed through scientific, professional, and patient associations, and compliance with the criteria was reviewed by the study coordinator team.

### 2.4 Discussion meetings

To compare perspectives, a similar discussion map including key topics was used for all discussion groups (Table S1). Neurologist‐specific topics, such as criteria guiding practitioners' decisions, were omitted in the non‐specialist groups. The discussions took place in Madrid (Spain) in spaces suitable for optimal interaction and free from potential sources of discourse bias (e.g., the presence of representatives from pharmaceutical companies). All discussions were moderated by an external expert in qualitative research who managed the time schedule, group members' participation, and the approach for all discussion map issues (Table S1). Prespecified questions were only brought into the discussion when the conversation did not flow naturally. All meetings were recorded.

### 2.5 Data extraction and analysis

**FIGURE 1:**
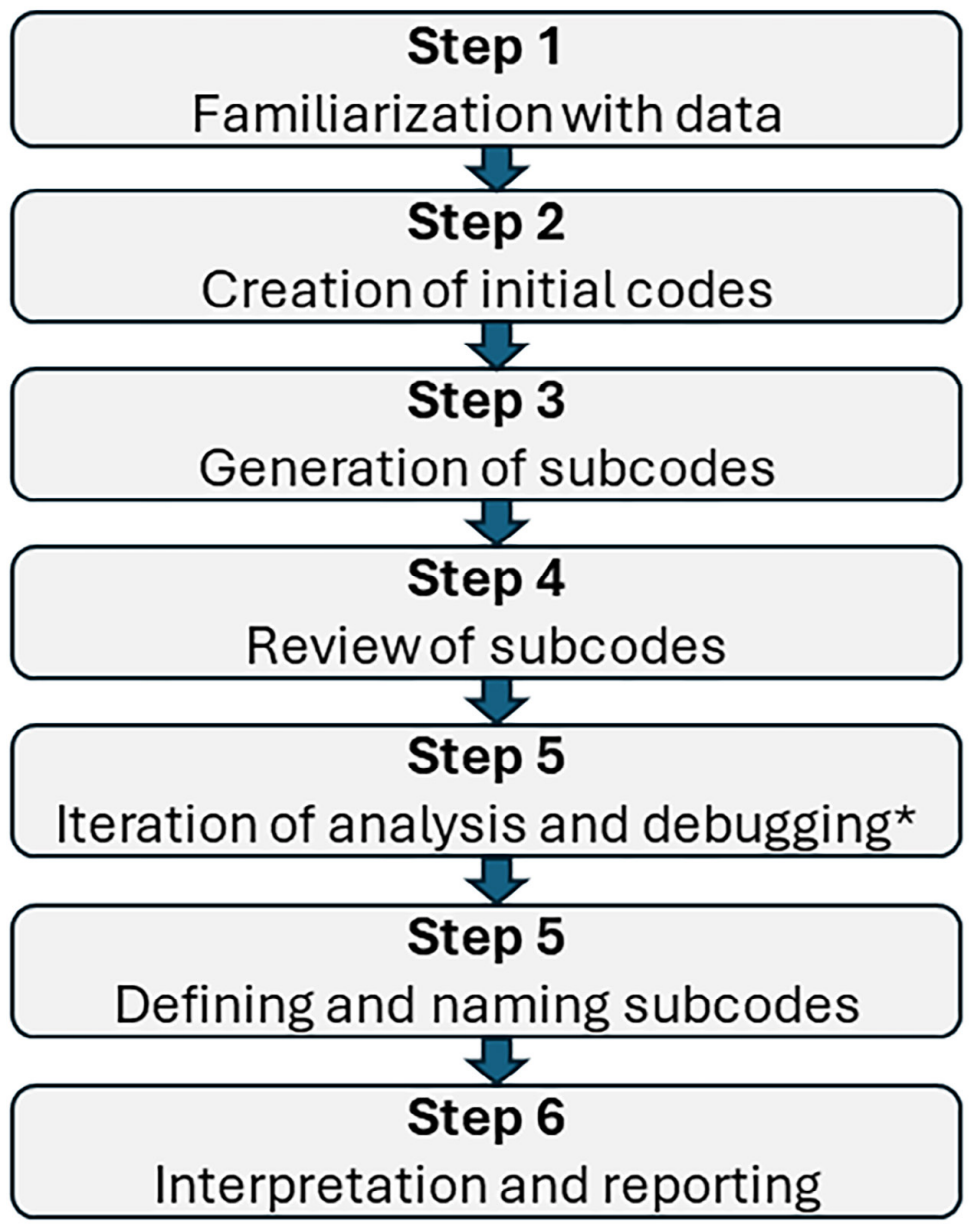
Flowchart of the thematic analysis process. *Repetition of analysis to ensure subcodes are not redundant and none have been omitted.

Audio recordings were transcribed by an independent company, revised by a methodology expert, and processed using Atlas.ti software (version 8.4). Transcripts were analyzed with the same software following standard procedures.^17^ Data were analyzed using two different approaches. First, a thematic content analysis was performed (Figure 1) by segmenting the discourse into aim‐focused relevant citations and discarding irrelevant ones. Productive citations were coded by “inductive analysis”^18^ according to the discussion map issues (Table S1; i.e., stages of the patient journey and barriers to their care). In a second step, we sub‐coded the information in each code using a constant comparative method following the phenomenological perspective (Grounded Theory).^19^ Thematic saturation^20^ was assessed from the number of citations and the number of connections for each code. Co‐occurrences between key steps and barriers were evaluated to identify the most relevant barriers to patient care.

## 3 RESULTS

### 3.1 Characteristics of study participants

A total of 36 neurologists, 10 nurses, 10 PWE, and nine caregivers participated in the study (*n* = 65; Table 1). The neurologists' subsample was larger than for other profiles to account for territorial diversity and to gather information on the patient circuit and management. The final distribution entailed four discussion groups of neurologists (8–10 professionals each) plus one group for each other category. Study participants were distributed in predefined profiles to maximize population representation. Female participants predominated in each profile group; neurologists younger than 55 years old and patients and caregivers under 65 years old predominated in their respective groups. Among neurologists, 33% (12/36) were epileptologists working in epilepsy units and 61% (22/36) were working at regional (secondary) hospitals. Among nurses, 60% (6/10) were general nurses and 40% (4/10) were nurse specialized in epilepsy. Only 11% of neurologists and 30% of nurses were working at Reference Centers, Services, and Units (RCSU). Most neurologists and nurses had more than 5 years of epilepsy experience. Among patients, 60% (6/10) were educated to secondary level (or equivalent), 60% (6/10) had >10 years of disease, 78% (7/9) had occasional or frequent epilepsy seizures, and 53% (8/15) had an age of onset over 18 years. Among caregivers, 56% (5/9) had received higher education and 67% (6/9) were caring for a patient with disease onset under 11 years; most caregivers were caring for a patient with >10 years of illness (75%; 6/8), cognitive impairment (71%; 5/7), or occasional epileptic seizures (56%; 5/9). Patients from county versus capital health centers were equally represented in the sample.

### 3.2 Thematic content analysis: Codes and subcodes

**FIGURE 2:**
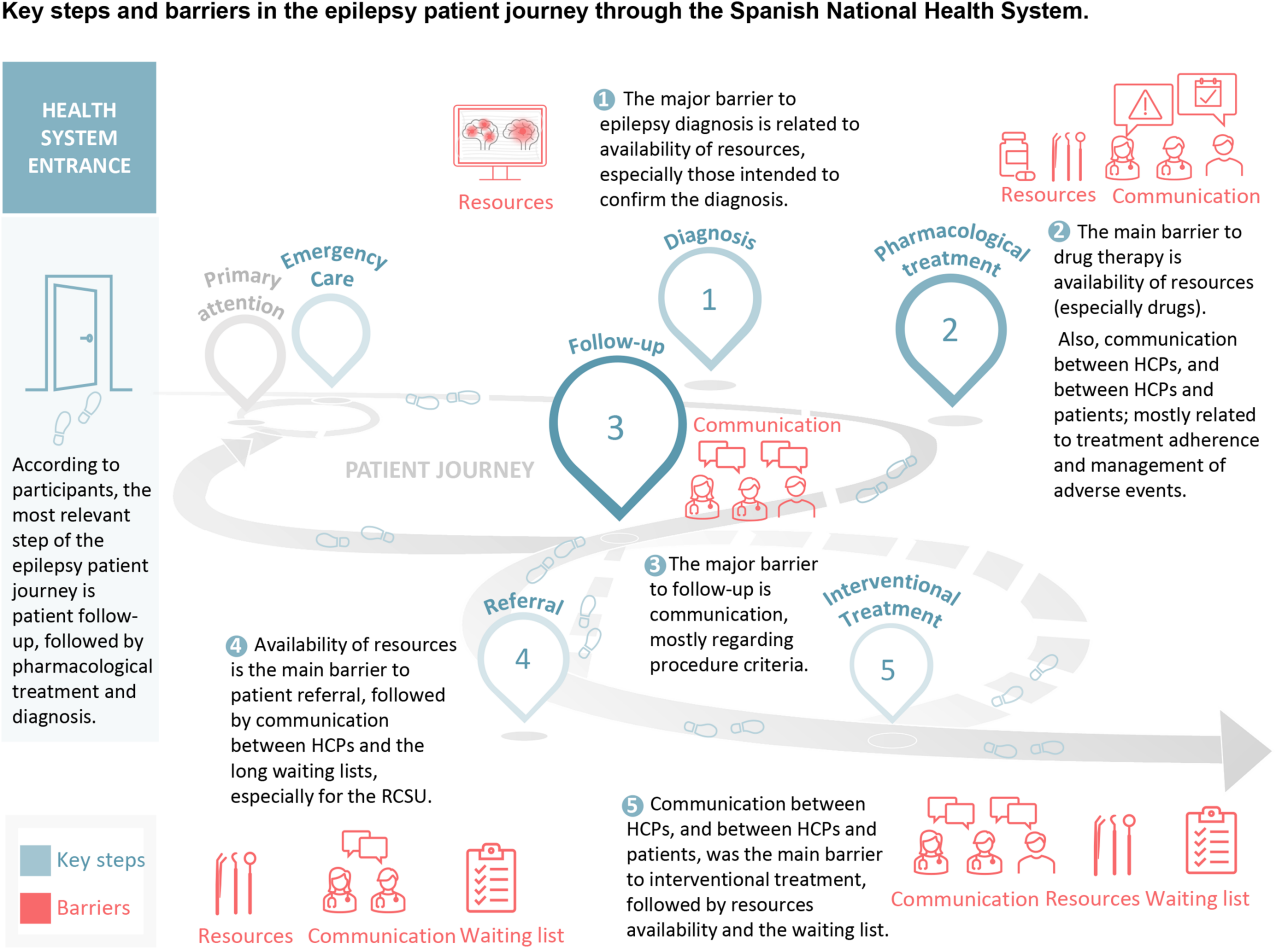
Key steps and barriers in the epilepsy patient journey through the Spanish National Healthcare System. Key steps (1–5) in the patient journey are shown in blue and barriers are shown in red. The relevance of key steps, elicited from group discussions, is reflected by label sizes. Patients enter the health system through primary and emergency care, and frequently return for prescription and follow‐up (primary care), and for treatment of uncontrolled seizures (emergency care). Patients also return from their reference hospital for follow‐up, after referral and interventional treatment. Communication between HCPs and between HCPs and patients is a barrier throughout the patient journey. HCPs, healthcare professionals; RCSU, Reference Centers, Services, and Units.

As described in Section 2, the discourse was discontinued into aim‐focused statements, assigned to group codes regarding the key stages and the barriers found. Non‐prespecified emerging codes (i.e. subcodes) were also identified. Table 2 shows the coding (codes/subcodes) and the definitions used in the thematic content analysis. The key steps identified were diagnosis, patient referral, drug therapy, interventional treatment, follow‐up, and visits to the emergency department (ED; Table 2, Figure 2). Communication, education, availability of resources, waiting lists, adverse effects, and variability in clinical practice were the barriers identified. In a similar analysis stratifying by healthcare providers (neurologists and nurses) and healthcare recipients (patients and caregivers), no remarkable differences in code distribution were observed (data not shown).

**TABLE 2:** Coding and definitions from the thematic content analysis.

Group	Codes/subcodes	Definitions regarding care to patients with epilepsy
Key steps	Diagnosis	How to perform the epilepsy diagnosis
	First contact	Who is responsible for the first contact with the patient for epilepsy diagnosis
	Diagnosis confirmation	Needs to accomplish an adequate epilepsy diagnosis
	Referral	Circuit and criteria for patient referral
	RCSU	Referral to Reference Centers, Services, and Units, epilepsy surgery units
	Epileptologists	Referral to an epilepsy unit or referring specialists
	General neurology	Referral to general neurology
	Other HCP	Referral to other health care professionals (HCP)
	Drug therapy	Management of epilepsy drug therapy and comorbidities
	Objectives	Objectives to achieve with the drug treatment
	Switch therapy	Decision making regarding change of treatment
	Prescription	Considerations for drug treatment choice
	Drug resistance	Management of drug resistance
	Interventional treatment	Interventional treatment (surgery, vagal stimulation)
	Objectives	Objectives to achieve with these treatments
	Criteria	Criteria to follow when performing these treatments
	Follow‐up	Patient follow‐up during epilepsy evolution
	Follow‐up periods	Follow‐up periods during epilepsy evolution
	Follow‐up criteria	Systems for patient follow‐up
	Professional in charge	Professional responsible for patient follow‐up
Barriers	Communication	Communication channels among stakeholders: HCP‐HCP, HCP‐other professionals, patient‐HCP, communications to general society
	Communication with patients	Relevant aspects of communication with patients
	Social awareness	Needs for greater awareness and knowledge of epilepsy at all levels
	Feedback among specialists	Communication among professionals involved in patient care
	Reports and informative documents	Materials to facilitate communication HCP‐HCP, HCP‐patient, general communication to society
	Ways of contact	The use of phone or email as ways of contact
	Education	Education needs in the field of epilepsy
	Lack of education	Educational deficiencies that impact on care of patients with epilepsy
	Education of other professionals	Educational needs of other professionals
	Available resources	Availability of tools and resources for suitable attention to epileptic patients
	Drugs	Drug availability
	Staff	Personnel needs for adequate attention to the patient with epilepsy
	Work‐up	Availability of electroencephalograms for diagnosis and/or follow‐up
	Schedule	Time available for a good performance of activities involving an adequate attention to the patient with epilepsy
	Waiting list	Waiting list for access to other services or resources needed (tests, interventional treatments, follow‐up consultations, or care by other professionals)
	Adverse effects	Adverse effects of treatments used for epilepsy control
	Variability	Negative impact of clinical practice variability in good management of patients

*Note:* The shaded rows are categories and the rest correspond to the sections included in each category.

Abbreviations: ED, emergency department; HCP, healthcare professional; RCSU, Reference Centers, Services, and Units.

### 3.3 Thematic saturation assessment

Thematic saturation,^20^ based on the data collected or analyzed to date, indicates that emergence of new themes and codes has reached a plateau, and further data collection or analysis are unnecessary to draw conclusions. We assessed thematic saturation by cross‐referencing data from the number of citations (theme relevance) and the number of connections across categories (density; Table 3). According to the number of citations, the most relevant step of the patient journey impacting the delivery of high‐quality care was follow‐up, followed by drug therapy and diagnosis (Table 3, Figure 2). However, the visits to the emergency department had the highest number of connections. Regarding the most relevant barriers to the delivery of high‐quality care for PWE, communication and availability of resources had the highest number of citations and communication had the highest number of connections (Table 3, Figure 2).

**TABLE 3:** Thematic saturation.

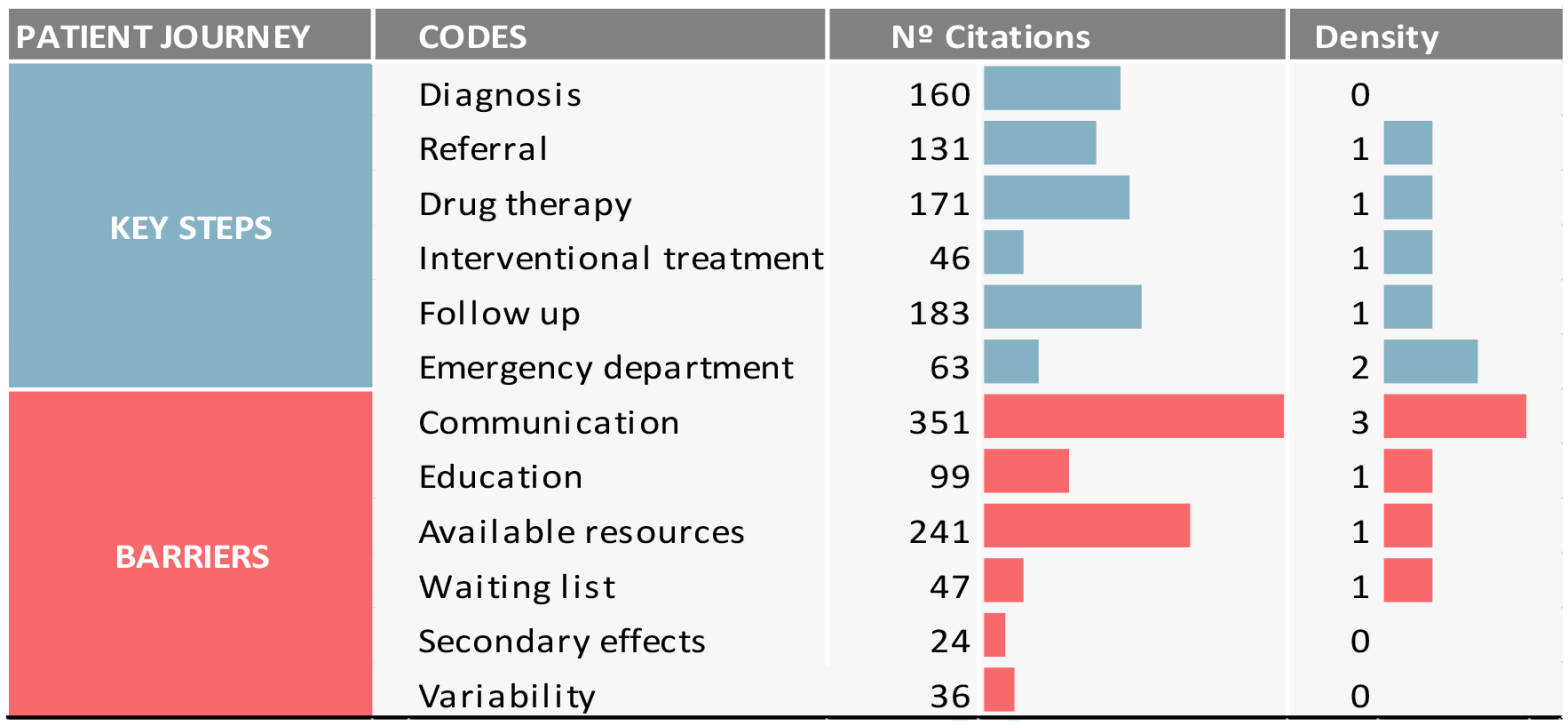

*Note*: Key steps (blue) and barriers (red) to the delivery of high‐quality care for patients with epilepsy are shown. Thematic saturation is reached when further data collection does not elicit new themes or issues for a specific population. The number of citations for each code gives an idea of theme relevance, and the density parameter quantifies the number of connections across categories.

### 3.4 Co‐occurrences

Mapping of co‐occurrences between key steps and barriers along the patient journey provides valuable information on the hurdles impeding adequate care delivery at each stage (Table 4, Figure 2). The main barrier to diagnosis was resource availability, particularly resources intended to confirm the diagnosis. Similarly, resource availability was the main barrier to patient referral, followed by communication between HCPs and the waiting list (especially for the RCSU). For drug therapy, the main barriers were resource availability (i.e., drug availability), and communication between HCPs and between HCPs and patients (mostly related to treatment adherence and management of adverse events). For interventional treatment, communication between HCPs and between HCPs and patients was the main barrier, followed by resource availability and the waiting list. The criteria to follow when performing treatments were considered the major hurdle for all of these issues. For HCPs and patients, the main barrier to patient follow‐up was communication, predominantly regarding who oversees follow‐up and how it should be done. The details of each co‐occurrence (i.e., key step and its main barriers for both professionals and patients) are displayed in Tables S2–S6.

**TABLE 4:** Co‐occurrences between the critical steps and the barriers in the epilepsy patient journey.

Barriers	Steps in the patient journey					
	DIAGNOSIS					
		DIAGNOSIS	First contact	Diagnosis confirmation		
	Communication	15	2	14		
	Education	19	9	12		
	Available resources	66	8	59		
	Waiting list	12	2	11		
	Variability	4	2	3		
	REFERRAL					
		REFERRAL	General neurology	Epileptologist	RCSU	Other HCP
	Communication	13	3	7	6	4
	Education	7	2	3	0	4
	Available resources	22	4	9	12	6
	Waiting list	10	5	4	8	1
	Variability	3	1	3	1	0
	DRUG THERAPY					
		DRUG THERAPY	Prescription	Drug Resistance	Switch therapy	
	Communication	27	17	1	7	
	Education	6	5	1	2	
	Available resources	53	33	16	5	
	Waiting list	5	3	1	1	
	Secondary effects	16	10	0	7	
	Variability	5	3	0	2	
	INTERVENTIONAL TREATMENT					
		INTERVENTIONAL TREATMENT	Criteria	Objectives		
	Communication	11	9	3		
	Education	2	2	0		
	Available resources	7	7	1		
	Waiting list	6	6	1		
	Variability	1	1	0		
	FOLLOW‐UP					
		FOLLOW‐UP	Follow‐up periods	Professional in charge	Follow‐up criteria	
	Communication	51	8	21	31	
	Education	10	1	9	1	
	Available resources	7	4	3	1	
	Waiting list	7	3	2	4	
	Variability	4	0	3	1	

*Note*: The gradation of numbers and colors correlates with the relevance of the barriers.

Abbreviations: HCP, healthcare professional; RCSU, Reference Centers, Services, and Units.

### 3.5 Emergency care and primary attention

Participants highlighted the emergency service and primary attention as the most frequent routes to diagnosis; entry into emergency care occurs for critically ill patients with seizures or when uncontrolled seizures occur (Figure 2).

### 3.6 Diagnosis

Diagnosis was the second key step for patient management, after presentation, and facilitates appropriate treatment selection to achieve best outcomes. For an adequate diagnosis: (a) the patient should be attended by a neurologist (preferably an epileptologist); (b) an encephalogram (EEG) should be performed by epileptology‐experienced professionals within a few hours after the seizure; and (c) a good anamnesis is a requirement (Table S2).

### 3.7 Drug therapy

The goal of drug therapy is to achieve seizure‐freedom for patients (which is not always possible), without secondary effects and emotional instability (Table S3). The main barrier to drug therapy was drug availability (e.g., due to frequent pharmacy shortages), particularly at the local level. Physicians highlighted a trend among pharmacists to switch prescriptions for generic drugs. In addition, patients must make unnecessary visits to keep their medication when a formulation is no longer valid, or the package doses do not match the prescription.

A barrier for drug‐resistant patients was considered to occur if the hospital pharmacy prioritizes economic criteria over patient benefit when switching/adding on treatment, even when supported by clinical data. Bureaucracy and management‐related problems were highlighted as additional barriers at hospitals.

Finally, treatment switch/addition is considered for patients not complying with predefined objectives (i.e., patients with uncontrolled seizures, unwanted secondary effects, or lacking emotional stability). HCPs highlighted how conflicting situations may arise due to inconsistent criteria for switching among professionals. Conflicts can occur due to lack of agreement between professionals from public versus private centers.

### 3.8 Follow‐up

From all patient journey steps, follow‐up was considered to have the greatest variability and discrepancy among the multiple options available. Absence of a time schedule and a follow‐up screen specific to each patient's characteristics was a hurdle to appropriate follow‐up (Table S4). There is also typically no designated person responsible for monitoring, and monitoring channels optimized for best follow‐up are not defined. Thus, communication between stakeholders involved in the care process was considered a major obstacle in follow‐up (Table S4, Table 4).

HCPs noted that there is no fixed rule for follow‐up intervals; annual frequency is the most commonly used, which may be considered reasonable for seizure‐free patients but is inadequate for those with persistent seizures. This is a particular issue in places without reference units for referral, or where existing units require patients to travel long distances. Participants noted that there should be a single person responsible for follow‐up to guarantee appropriate attention for the patient. Finally, failure of communication between primary and secondary care, or between reference centers and RCSU, was a barrier to follow‐up; there is also an issue of flow of information collected through electronic clinical history systems from different hospitals (Table S4).

### 3.9 Referral

Barriers to specialist care included a lack of professionals and long waiting lists (Table S5). HCPs noted that the more specialized the care needed, the more acute the problem becomes, with access to RCSUs very limited. HCPs highlighted three major reasons for this: a paucity of specialized centers; difficulty in identifying and following candidates; and long waiting lists, both for attention and follow‐up. Improving communication between professionals after referral was highlighted as a key goal. Finally, collaboration with other professionals was considered important for patient management (e.g., psychiatrists and psychologists), but access to these professionals is limited and long waiting lists are considered a deterrent.

### 3.10 Interventional treatment

Physicians noted that patients with drug‐resistant epilepsy are candidates for epilepsy surgery, and there are established criteria to consider surgical options (Table S6). Physicians highlighted the aim of surgical treatment to improve patients' quality of life, but noted that achieving seizure‐freedom or freedom from medication is unrealistic. However, relevant improvements can be achieved in selected patients, and they should be informed of the existing options. In addition to referral limitations (i.e., limited access to monitoring and specialist units), a major hurdle to interventional treatments was considered to be limited delivery of information to HCPs and patients (Table 4).

## 4 DISCUSSION

To our knowledge, this is the first qualitative study on epilepsy care management in which four major care stakeholders (neurologists, nurses, patients, and caregivers) shared their experiences and opinions within discussion groups. This has allowed us to provide a comprehensive overview of the epilepsy patient journey through the Spanish NHS and to identify areas of improvement that can lead to future standardized recommendations for care. Emergency care, diagnosis, drug therapy, follow‐up, referral, and interventional treatment were identified as the key steps in the patient journey. Follow‐up was identified as the step with greatest potential for improving care, followed by drug therapy and diagnosis. When assessing thematic saturation, the highest number of connections was for emergency department visits, suggesting that the quality of emergency care has implications at every step of the patient journey.

We detected several barriers to the delivery of high‐quality care at each stage of the patient journey: resource availability is a major barrier for diagnosis and for several additional steps; lack of professionals and specialized centers, together with long waiting lists, are key barriers for patient referral; and drug availability is an issue for drug therapy. Based on thematic saturation, communication (between HCPs and between HCPs and patients) had the highest number of connections, consistent with its significant impact on every step of the patient journey. This is particularly relevant with regard to adherence and management of adverse events during drug therapy, and for several aspects of follow‐up (i.e., ensuring a specified professional to coordinate follow‐up, and choosing the best procedure and time interval to use).

Barriers to the delivery of high‐quality care identified in our study impact on several key issues of epilepsy management, including adherence to appropriate treatment in the era of polypharmacy, and the drive to achieve seizure freedom.

Treatment adherence and management of adverse events during drug therapy are likely impacted by communication issues between HCPs and between HCPs and patients, drug availability, and a lack of professionals and specialized centers. Our findings align with other studies on current epilepsy management in Spain and other countries. A study of psychological unmet needs in patients from the United Kingdom, France, Germany, Italy, and Spain receiving polytherapy identified a lack of information, support, and resources as major barriers to care both pre‐ and post‐diagnosis.^21^ A lack of adequate information and shared decision‐making between neurologists and patients, and unclear criteria for prescribing were identified as major hurdles during treatment. The study also detected non‐adherence behaviors at this stage, with a negative impact on outcomes. During follow‐up, patients highlighted inadequate support, lack of communication, and low social awareness of epilepsy leading to stigma and discrimination.

Managing expectations related to side effects of a new treatment may alleviate anxiety and reduce the risk of nonadherence and seizure recurrence, according to a study on the mental burden of PWE.^22^ The researchers indicated the need for effective education programs to raise awareness and understanding of these issues among HCPs, and support and education initiatives for patients. This aligns with an investigation highlighting the deleterious effects of non‐adherence to ASMs, which drives poorer seizure control.^23^ The authors indicated the need for patient‐centered treatment plans and thorough patient counseling regarding the treatment process to boost self‐administration practices and symptom control.^23^

While seizure freedom is not always achievable with ASMs, evidence suggests that ASM polytherapy is an effective approach in many patients.^24^ However, uncontrolled patients can encounter several barriers to new and potentially effective treatment options, including: hospital pharmacies prioritizing economic over clinical criteria when prescribing new treatment; therapeutic nihilism among attending HCPs; and low expectations of success among patients. A lack of communication, resources and training within the healthcare system exacerbates these issues. Furthermore, with no fixed schedule for follow‐up, uncontrolled patients may experience delays in switching to, or adding, a new medication, which can potentially lead to recurrence of seizures and reduced quality of life. A recent Australian qualitative study has explored the experiences of patients with newly diagnosed epilepsy as they await effective seizure control. The study highlighted a need among patients to restore a sense of control in their lives and found a willingness among them to support technology systems that assist in the selection of effective medications.^25^ In Spain, Villanueva and collaborators^26^ identified relevant unmet needs for patients with drug‐resistant epilepsy, including specific treatment protocols, specific ASMs with improved effectiveness and safety profiles, and increased referrals to specialized epilepsy units to promote early diagnoses.^27^ The researchers highlighted issues of underdiagnosis and a lack of reliable epidemiological data in Spain.

Finally, a systematic review has shown how both high‐ and low‐income countries experience poor healthcare availability, access issues, and lack of health information which contribute to unmet needs in PWE.^28^ The study highlighted how lack of health services, long wait lists, uncoordinated care, and poor access to health information were prevalent both in the United States (US) and in countries with a universal healthcare system.

A strength of our study is that we provide new insights into epilepsy care in Spain by collecting, for the first time, feedback from four care stakeholders to integrate different perspectives on the management of PWE. A minimum of participants within the groups encouraged productive discussion, with at least one representative of each prespecified profile per group. For a comprehensive assessment of the patient journey and management, the number of participating neurologists was four times higher than for other profiles. The qualitative methodology is a valuable tool for obtaining real‐world insights into epilepsy care, to answer complex questions, and to identify best practices for implementation.

As an exploratory approach, our study has several limitations. Qualitative methodology attempts to answer “how” and “why” questions without reliance on quantitative measurement or statistical analysis.^29^ Its value depends on the research subjects, the quality of conversations, and the adequacy of the sample size. The number of groups was limited, and subgroups of individuals may not have been represented in the sample. In particular, there was a higher representation of neurologists compared with patients/caregivers, although no representation from emergency physicians or primary care physicians. This may have impacted on the frequency of specific key areas/barriers identified by the groups. Also, results may differ depending on the chosen criteria to prespecify patient characteristics (e.g., pediatric population, social level, and region). In this regard, though education level was included as a criterion for patient characteristics, conversations might vary depending on the social level of patients/caregivers, bearing in mind that the incidence of epilepsy in high income countries is highest among poorer people.^30^

There are several avenues for further research. First, the stakeholder profiles could be broadened to include categories involved in managing the pathology and the patient journey indirectly, such as pharmacists, psychiatrists/psychologists, social workers, health managers, and health administration representatives. This would provide a multidisciplinary and multisectoral analysis of the patient journey. Additionally, stratifying by autonomous communities may be useful in identifying regional differences in epilepsy care.

## 5 CONCLUSION

In conclusion, we provide a comprehensive overview of the epilepsy patient journey through the Spanish NHS and identify key steps and barriers to the delivery of high‐quality care that can be used to inform future management recommendations. Ultimately, homogenization of the management and care of patients throughout the national territory is an unmet need that is yet to be addressed in the Spanish NHS. While a regional approach could have an easily actionable starting point and a straightforward and short‐term impact on patients, we should aspire to create an improved national egalitarian healthcare system. Barriers to the delivery of high‐quality care highlighted here can be used to construct a roadmap for the design and implementation of specific actions led by health administrations to improve the care and quality of life of PWE with a chronicity perspective.

## AUTHOR CONTRIBUTIONS

Ángel Aledo‐Serrano, Juanjo Poza, María Palanca, FEDE, Paloma Pérez‐Domper, and Milena Gobbo contributed to the conception and design of the study and to acquisition of data. Milena Gobbo analyzed the data. All authors discussed the results, revised the first draft, and contributed to the final manuscript. All authors have read and approved the submitted version of the manuscript and accept the responsibility for its content.

## FUNDING INFORMATION

This research was funded by Angelini Pharma.

## CONFLICT OF INTEREST STATEMENT

JJP has received honoraria for advisory, educational activities, and/or research funds from Angelini Pharma, GlaxoSmithKline, Bial, Eisai, Jazz Pharmaceuticals, Neuraxpharm, Sanofi, and UCB Pharma. MG has received honoraria for this project from Angelini Pharma. MC has received honoraria for educational activities from Angelini Pharma. FEDE has received funding from Angelini Pharma, Biocodex, Eisai, Health in Code, Jazz Pharmaceuticals, Neuraxpharm, Nutricia Danone, Sanofi, Takeda, and UCB Pharma. PPD is an employee of Angelini Pharma. AAS has received honoraria for educational activities and/or research funds from Angelini Pharma, Bial, Blueprint Genetics, Eisai, Health in Code, Jazz Pharmaceuticals, Neuraxpharm, Nutricia, and UCB Pharma. We confirm that we have read the Journal‘s position on issues involved in ethical publication and affirm that this report is consistent with those guidelines.

## Supporting information


Table S1

## Data Availability

The original contributions generated for this study are included in the article or its Supplementary Materials. Further inquiries can be directed to the corresponding author.
