# Supplementary material for: Key steps and barriers in the journey of patients with epilepsy through the National Healthcare System in Spain: The EPIPASS qualitative study
Source: Epilepsia Open. 2024 Jul 4;9(5):1731–44. doi: 10.1002/epi4.12984 (PMC11450596; doi:10.1002/epi4.12984)
Supplement: Supplementary file 1 — Table S1 [file EPI4-9-1731-s001.docx]

**Supplementary Table S1.** **Discussion map.**

| **Steps in patient journey through the National Healthcare System (NHS)** |
| --- |
| - Highlight the stages of the patient journey through the NHS, from the first consultation/emergency related to the disease or the first neurology evaluation to obtaining the diagnosis and accessing optimal treatment - For each stage, what is the current situation and what would be the ideal situation? - What are the barriers to achieving the ideal situation? |
| **Criteria to pass from one stage to the next** |
| - For each of the following questions, please describe the current situation, the ideal situation, and any barriers you see to achieving the ideal situation:   - What data and material/human resources are needed to establish the diagnosis of epilepsy?   - What criteria are taken into account for referral to a specialist (epileptologist)/referral centre?   - What criteria and objectives are pursued in terms of:     - Starting pharmacological treatments     - The choice of treatment     - Removal and replacement of treatments     - Combining treatments     - Assessing treatment effectiveness     - Assessment of adverse effects (tolerability)   - What criteria and objectives are pursued when using non-pharmacological treatments:     - Epilepsy surgery and/or palliative techniques (e.g., vagal stimulation, deep brain stimulation)     - Objectives to be achieved with these treatments   - How is treatment tracked?   - What role do clinical practice guidelines play? |

**Supplementary Table S2. Barriers to diagnosis of patients with epilepsy, consequences, and potential solutions.**

| **Subcodes** | **Barriers** | **Citation examples** | **Consequences** | **Possible solutions** |
| --- | --- | --- | --- | --- |
| Diagnosis Confirmation | Lack of neurologists and lack of training of general neurologists at the ED. | *● Group 1 - Physicians. The neurologists on duty at the hospital are not epileptologists in most cases. However, I think a general neurologist on duty is able to diagnose a first seizure.* | Delay of epilepsy diagnosis. | Design a protocol to ensure availability of neurologists at the ED or to quickly refer a patient to a neurologist. |
|  |  | *● Group 2 - Physicians. I think there are several barriers. The first one is when the patient presents with their first seizure, the lack of knowledge or time for the personnel assisting the patient, including neurology residents or some general neurologists.* |  |  |
|  | Unavailability of EEG equipment. | *● Group 1 - Physicians. ... But the basics are missing in 95% of the hospitals, which includes 24-hour availability of electroencephalograms (EEGs).* | Delay of epilepsy diagnosis. | Ensure equipment availability to perform EEGs 24 hours a day at any hospital or neurology unit.   Educate neurologists to perform and interpret an EEG. |
|  |  | *● Group 2 - Physicians. We are negotiating with neurophysiology to buy an electroencephalogram, but we find their denial alleging professional intrusion. We are struggling to buy the apparatus so that resident physicians, and we ourselves, get some training to manage the device in an emergency.* |  |  |
|  |  | *● Group 7 - Carers. What frequently fails is not doing the diagnosis correctly as tests are not performed. We struggle for a few years with an idiopathic generalized epilepsy, without knowing the real etiology and no tests are performed. There is an SEN-approved protocol, but it's not applied.* |  |  |
|  | Lack of time for an adequate anamnesis. | *● Group 3 - Physicians. Listening to the patient is crucial, which is the basis for diagnosis. What they tell you can give you the clue; the basis for me, for a good diagnosis, is the time listening properly to the patient.* | Delay of epilepsy diagnosis. | Increase the time devoted to the first visits. |

**Abbreviations:**; EEG, electroencephalogram; GP, general practitioner; SEN, Spanish Neurology Society.**Supplementary Table S3. Barriers to drug treatment of patients with epilepsy, consequences and potential solutions.**

| **Subcodes** | **Barriers** | **Citation examples** | **Consequences** | **Possible solutions** |
| --- | --- | --- | --- | --- |
| Prescription | Drug shortages at the pharmacies. | *● Group 2 - Physicians. Drugs are lacking due to shortages. ● Group 7 - Carers. The option in the end is going to many pharmacies until you find it. The problem is in small places; not for me, because I am a driver and visit many places.* | Patients don't get access to the medication that best suits their needs. | Control stocks at the pharmacies to guarantee drug supplies to the patients. |
|  | Neurologists' prescriptions are switched by the pharmacists for generic drugs. | *● Group 4 - Physicians. There are many pharmacies in Pamplona, and (drugs) are usually changed; it's a different way of working, we have to insist a lot to the patient on taking that drug with that specific name and ask for it, because it is important in epilepsy to avoid drug changes every time they go to a pharmacy. They are given a drug from a laboratory one day, and a different one the following month. They are more irregular each time, they are not equal, and in order to have a patient controlled, their medication shouldn't be changed every month that they go for a new package.* |  | Elaborate documents justifying the need to maintain prescriptions according to the neurologist's criteria. |
|  | The visits for patients do not always match prescriptions. | *● Group 1 - Physicians. It cannot happen that a patient runs out of medication because the system only allows you to prescribe for 6 months, and you have made the next appointment in 8 months. ● Group 6 - Patients. I used to have X, with a monthly administration, and I don't know if it's an informatic issue that the same pill of a different dose cannot be provided; it provides me a pill one day and another one the following day, and I have to call the doctor every 15 days for a new recipe.* | Patients have to make unnecessary physician consultations. | Allow recipe validity until the following control visit.  Create drug packages with medication doses matching potential prescriptions. |
| Drug resistance | The hospital pharmacy does not facilitate the off-label use of the drug. | *● Group 4 - Physicians. Within the treatments we prescribe, we find a major problem with the hospital pharmacy. They are very strict when prescribing a foreign medication not approved in Spain, and it's more difficult. ● Group 4 - Physicians. We struggle to have the last endovenous drugs available at the hospital. We don't have them, though we would like to. ● Group 4 - Physicians. When one (drug) is much cheaper, you have no access to the other one; but sometimes a patient doesn't respond, has an allergy...we should have the other one, but we haven't. I think this happens in all hospitals, which follow the economic criteria. ● Group 4 - Physicians. Pharmacists ask us to show them studies comparing the two drugs proving that one is equal or better than the other one, but it is hard to tell because evidence is lacking. This is how they support their arguments.* | Patients cannot buy the medication that better suits their needs, or have to make unnecessary visits. | Provide documents justifying the needs based on scientific evidence. |
|  | There is a lot of bureaucracy and access delays to this type of medication. | *● Group 3 - Physicians. The other conditioning factor and the great limitation for hospital prescription is the bureaucracy; the months you are waiting for; I think that the process is halted at the pharmacy, at the management office... so you end up prescribing another drug. ● Group 3 - Physicians. One limitation we have is that in the midterm year you are told “you have 23% of therapeutic novelties, when it should be less than 20%, and we'll meet again in 3 months”, so that conditions you to prescribe less; if you have an interim contract, you try to comply, but if you are a permanent employee, you don't care.* | Neurologists find hurdles to use medications that could improve patients' health status. | Reduce bureaucracy hurdles for prescription. |
| Treatment switch | It's not clear who takes the decision about the medication when several professionals are involved in patient management | *● Group 1 - Physicians. There are people going to private care, they go to the hospital in their community, and also come with us. Many times I have a passive role because, if you have your medication switched 15 days ago from somewhere else, what can I do? ● Group 2 - Physicians. There are many variables to consider: age, other diseases, concomitant treatments, epilepsy type, etc. Most patients are referred from emergency care with some seizure that may be compatible or not with a seizure; they come on a drug, that may be endovenous, normally. They are referred to my office, and you have to decide what to do: continuing with that therapy or suggesting a change, but this means getting the patient into trouble; the patient may think "if I was prescribed this medication and I feel well, and this physician is suggesting a change that can make me worsen or cause new secondary effects...” There is a conflict in the end when a patient presents with a diagnosis and ongoing treatment.* | Different prescriptions provoke conflicting situations. | Find ways to integrate public-private attention in a standardized way. |

**Abbreviations:** i.v., intravenous.

**Supplementary Table S4. Barriers to follow-up of patients with epilepsy, consequences, and potential solutions.**

| **Subcodes** | **Barriers** | **Citation examples** | **Consequences** | **Possible solutions** |
| --- | --- | --- | --- | --- |
| Follow-up period | There are no clear standardized schedules for patient follow-up adjusted to their needs. | *● Group 1 - Physicians. In some cases you may give an appointment in 3 months, but it is finally scheduled in 12 because there is no room, despite the need to see that patient in 3 months. It (a standardized follow-up schedule) would facilitate the patient being seen more frequently by epilepsy specialists. ● Group 2 - Physicians. We have a refractory epilepsy unit, 2 consultation offices a week, and I would need at least 4 a week to have the patients controlled as I would like to. I have room for 12 refractory patients and I end up seeing 16 badly, rushing and short of time. I always have the feeling of not providing the adequate care I should be delivering. ● Group 4 - Physicians. The follow-up waiting list is a flaw of the system. It depends a lot on each specialist. In my case, there is a very long waiting list, I would say a year, but it could be two, and that's terrible. Patients complain a lot; broadly speaking, we see them annually.* | Patients usually have appointments they don’t need, and need appointments they don’t have in a timely manner. | Establish standardized criteria for the patients’ follow-up following stratification criteria. |
| Follow-up system/ criteria | Follow-up can be on-site or off-site. Online or telephone follow-up are not developed enough to take advantage of their benefits, and still present inconveniences. | *● Group 1 - Physicians. Having a contact number and talking to a nurse who may ask you later, that provides a lot of confidence to the patients.  ● Group 2 - Physicians. I have a contact email, and sometimes they send you a message each half an hour, 20 in a week, until you tell them this resource is only for emergencies. ● Group 3 - Physicians. Sometimes we connect on WhatsApp, but it’s not the right thing to do because some data or certain things are lost, and, in the end, you don’t even know what you have told them. ● Group 7 - Carers. In this sense, it depends on the physician because if the person is accessible, it makes your life easier; getting quick access is quality of life for the patient and the carer. Since we have access to the neurologist, if we want, we can go less to the hospital, call less and worry less. We know that if we have a problem, we can call and will get the call back as soon as they are available. The doctor is always open; when we go to hospital, they would attend us if available, and we don’t need to keep going every X months to consultation because those 6 months can be ok for now, but not for later on. That is quality of life. ● Group 2 - Physicians. The lack of time for consultation is also coming up in the discussion, and this should not mislead our supervisors or management teams. Teleconsultation is good for the patient because they don’t need to commute, spend money and waste time, but for us, it means the same time as onsite consultations. An onsite consultation cannot be replaced by 3 e-consultations because it takes the same time or even more, because you call the patient, their mother, their teacher… To sum up, this cannot be the excuse to increase the number of patients.* | Professionals and patients cannot benefit from a well-designed, efficient, remote attention system. | Adopt off-site attention routes that are useful and practical for patients, professionals, and management teams. |
| Professional responsible for monitoring | It's not clear who should be monitoring at each time. | *● Group 2 - Physicians. For the patient-physician relationship in chronic diseases, it's very important that the patient is always seen by the same physician. One thing that happens to me is that there are a few of us alternating in the epilepsy consultation office at the hospital. If I am on duty and a colleague is on consultation, the patient would call me the following week. There must be continuity to maintain a relationship.  ● Group 7 - Carers. Neurologists of the National Health System shouldn't have temporary contracts that change every 6 months; you have to tell them your life and start again from scratch.* | Patients do not know who they should contact for the follow-up, because there is not a single person responsible for that. |  |
|  | Communication between professionals responsible for patient monitoring is deficient.    There lacks coordination between primary and specialized attention of patients with epilepsy. | *● Group 4 - Physicians. Regarding communication with a primary care provider through an online interconsultation - one can have a patient relatively controlled, but who suddenly has a seizure, goes to the primary care provider, and through an online interconsultation they can ask you what to do with that patient. You can tell them anything through that interconsultation, helping to solve many things; sometimes it even helps to make an earlier appointment for the consultation scheduled for 3 months later. When a patient has been controlled for many years with a medication, any change could also be managed through the interconsultation.  ● Group 5 - Nurses. Both care providers must be equally involved for things to work, each of them with their knowledge and specialization. ● Group 5 - Nurses. The second problem is the non-existent coordination between primary and specialized attention. They are on different planets, and we are seeing the same patient; and as we ourselves are not capable of saying “let's coordinate ourselves, plan something in common”, then we keep on finding that barrier. ● Group 1 – Physicians. There are patients coming from another community, strongly drug-resistant, and that makes me anxious because I don’t know how to contact their physician; it’s not having anyone close to the patient who can supervise what I do. ● Group 3 - Physicians. Communication is crucial. If I've known a patient at the office for 10 years, and you send them to a unit in another autonomous community, communication is lost to a great extent despite making a comprehensive report. ● Group 3 – Physicians. Considering that we are not very prone to make reports, and also the great mobility of the population from one community to another, it would be reports or computerized history. That is a flaw of our system, that you cannot know what the internist treating the patient thinks. ● Group 5 - Nurses. It's not so much a communication issue, as the information systems between management teams, both in primary and hospital care, and even in different hospitals. Each one uses a different informatics system, so if the patient doesn't bring the report provided at the emergency room or from the private physician to the primary care consultation, you have nothing. The physician may fill in the history in their program at the hospital, but the primary physician cannot access the patient history.* | The lack of a single person in charge for the follow-up generates gaps and contradictions between professionals. | Improve communication between professionals responsible for follow-up of patients: - between primary and specialized attention - between general neurology and specialized centers or RCSU - having a reference person to coordinate the patient follow-up (e.g. nurse) |

**Abbreviations:** RCSU, Reference Centers, Services and Units.

**Supplementary Table S5. Barriers to referral of patients with epilepsy, consequences and potential solutions.**

| **Subcodes** | **Barriers** | **Citation examples** | **Consequences** | **Possible solutions** |
| --- | --- | --- | --- | --- |
| Referral to specialized neurologists (RCSU) | Lack of clear referral protocol | *● Group 2 - Physicians. Patients are referred to the surgery units and some are referred too late, more delayed patients, more complicated, with less indication for surgery. I have the feeling that many of them are lost on the way, and would be good surgery candidates if they would have been taken earlier, providing them with a much better prognosis and quality of life improvement. ● Group 3 - Physicians. Here is the bottleneck - these patients may have taken 17 years to be monitored, and they've been 17 years with a wrong diagnosis; some are not seizure cases; and others might be surgery cases, but when they get to be monitored, there is so much affectation that surgery is not going to improve their quality of life.* | Patients who could benefit from specialized attention are referred too late or not referred. | Establish referral protocols, or measures to implement those existing ones. |
|  | Impossibility of monitoring to identify candidates for surgery referral | *● Group 4 - Physicians. I work at a small hospital and I have no neurology on-calls. I don't have an electroencephalogram, so channelling these cases is hard.* |  | Facilitate patient monitoring. |
|  | Long waiting lists for RCSU centers and for monitoring | *● Group 2 - Physicians. I work in an epilepsy surgery center and we realize that patients come late; all the processes are very slow and after trying the drugs, after seeing that one is failing, then another one, years go by; and when you say, this is a surgical case, maybe 6 years or more have gone by. With the resources in the past, it took 20 years to get to the epilepsy surgery unit. Now it is less, but there are just a few units in the country and also many waiting lists. ● Group 4 – Physicians. Centers with more capability to attend more patients. More capability is more cost-effective because resources will be divided among more RCSUs, having to invest in everything again. However, if you are in one RCSU, you may have a lot of equipment that will be helpful at the same time. What you need is more personnel doing the same things. This is more cost-effective.* | Surgery is not available in a timely manner or in the right way in cases where it would be useful or necessary. | Increase the number of RCSU centers, or improve their performance by providing them with greater capabilities. |
|  | Lack of communication between professionals at referral | *● Group 1 – Physicians. If I have a patient referred from a physician – I miss having direct contact with that physician and keeping them informed with reports we can share and discuss. I would prefer having feedback from those physicians. It would be interesting to call them and tell them what we have seen, informing them straight away.* |  | Facilitate interaction among professionals (e.g. online meetings). |
|  |  | *● Group 3 – Physicians. Since the coronavirus, we are doing more online meetings, and we are trying to include referral agents. The referral person is the one who knows that patient more than yourself, and we try to include them in the meeting when taking the decision of whether to perform surgery or not. We are doing all surgery meetings with this system.* |  |  |
| Referral to other healthcare professionals (psychiatrist, psychologist, physiotherapist, social worker) | Not enough professionals for adequate epilepsy management | *● Group 5 - Nurses. Access is hard in my case. It's a middle-sized hospital with few professionals and recently incorporated to the health system; the problem is the unlimited waiting list, and the patients will enter with seriously severe conditions. The professionals can be contacted but they cannot take the patients. This will be easier when interaction with these professionals is normalized as part of the treatment.*  *● Group 6 – Patients. It is necessary for the neurologist to tell you the first time, ‘look, how are you feeling, and if not, we can refer you to a psychologist or psychiatrist.’ I went on my own and it is necessary due to the medication we take.* | Neurologists cannot refer patients to these professionals when needed. | Create a repository of public and private resources for referring patients in need of assessment or treatment delivered by other professionals. |
|  | Access to these professionals, when available, is complicated | *● Group 4 - Physicians. In our case, the neuropsychologist can only be requested by our colleague who attends to dementia cases. For everything else, they are not included in the service offered. ● Group 6 – Patients: After a seizure you have many contractures. After a tonic-clonic crisis, your back is terrible. I go to a private physio.*  *● Group 7 - Carers. Have you got access to these professionals? Psychologists, physiologists...Access to a private neuropsychologist is even more difficult, with just one per region. I didn't even know they exist.* | Patients have no access to these professionals who might improve their health status and quality of life. | Improve the systems of referral to other professionals. |

**Abbreviations:** RCSU, Reference Centers, Services and Units. **Supplementary Table S6. Barriers to interventional treatment of patients with epilepsy, consequences and potential solutions.**

| **Subcodes** | **Barriers** | **Citation examples** | **Consequences** | **Possible solutions** |
| --- | --- | --- | --- | --- |
| Criteria | Difficulties when referring patients for surgery, with access hurdles to: - monitoring (for candidate selection) - specialized units (long waiting lists) | *● Group 1 - Physicians. A patient complying with drug-resistant epilepsy criteria must be sent to the refractory epilepsy unit, where you can evaluate the surgery option. They must have a clear adhesion or epileptogenic area that is resectable. So the first step is having a drug-resistant epilepsy, and from there studying the case if resources are available.* | Patients who could benefit from surgery are not referred.  Surgery is not always available in a timely manner or in the right way in cases where it would be useful or necessary. | Reduce the monitoring waiting list.  Reduce the waiting list for interventional treatments. |
|  | Poor communication between general neurologists and professionals working at the surgery units. | *● Group 3 - Physicians. I don't know what has happened to the patient until they come to my office and they tell me that they have undergone surgery. I don't receive any report if they don't bring it personally. As we are different communities, we don't have access to it.* |  | Improve communication between specialized units and the referring professionals. |
| Objectives | Lack of information for patients about the options. | *● Group 1 - Physicians. Sometimes you have to be frank to the patient and tell them that the objective of vagal stimulation is not getting seizure-free, but reducing their seizure frequency when you prescribe a drug; you don’t tell them that there are cases of patients becoming seizure free on the eighth drug. ● Group 2 - Physicians. There is no epilepsy surgery unit in my centre, but there is one where I got trained. My experience was varied and depended on the patient. Many of them would respond to surgery and treatment would go well. We always had to inform patients that they wouldn't get seizure-free; that couldn't be guaranteed with surgery, and they wouldn't be free of drugs either. This is something they used to assume when undergoing surgery. ● Group 4 - Physicians. Improving their current quality of life with drug treatments would be the main objective. Improving quality of life is everything, not only controlling the number of seizures, but having more odds to enjoy sports, not having secondary effects with the medication... ● Group 7 - Carers. Be informed about surgery - that subject has not been brought up yet. We still haven't been suggested any epilepsy surgery; they fall short when it comes to informing us about epilepsy surgery.* |  | Inform patients about these surgery options. |
|  | Lack of training of nurses about these procedures. | *● Group 5 - Nurses. Do any of you have any knowledge of the surgery option in epilepsy management? No. No. We do, but in just a few cases, and the nursery does not have any role. A circuit could be implemented where the patients were informed about all the things that are going to happen.* |  | Educate nurses on the procedures that may improve management of the referred patients to the surgery units. |
